# New trends in cell death: overview of the International Society of Cell Death (ICDS) annual meeting in Johannesburg South Africa (May 19-22, 2023)

**DOI:** 10.1186/s12964-024-01534-9

**Published:** 2024-03-14

**Authors:** Marianne J. Cronjé, Varsha Gadiyar, Jerry Edward Chipuk, Tugba Bagci-Onder, Dmitri Krysko, Raymond B. Birge, Richard A. Lockshin, Zahra Zakeri

**Affiliations:** 1 School of Molecular & Cell Biology, University of Witwatersrand, Johannesburg, Gauteng South Africa; 2 Department of Microbiology, Biochemistry and Molecular Genetics, Center for Cell Signaling, Rutgers New Jersey Medical School, 205 South Orange Ave, , Newark, NJ 07103 USA; 3 Icahn School of Medicine at Mount Sinai, One Gustave L. Levy Place, New York, NY 10029 USA; 4 Koç University School of Medicine, Istanbul, Turkey; 5 Cell Death Investigation and Therapy Laboratory, Anatomy and Embryology Unit, Department of Human Structure and Repair, Faculty of Medicine and Health Sciences, Ghent University, Ghent, 9000 Belgium; 6 Cancer Research Institute, Ghent, 9000 Belgium; 7 Department of Biological Sciences, St. John’s University, Jamaica, NY 11439 USA; 8 Department of Biology, CUNY Queens College, Flushing, NY 11367 USA

## Overview and meeting theme

The International Cell Death Society (ICDS) Annual Scientific Meeting was held i

n Johannesburg, South Africa on May 19–22 (https://celldeath-apoptosis.org) with “New Trends in Cell Death” as the main meeting theme. This was the 29th anniversary of the ICDS Society, a group that was started in 1994 at Rockefeller University as ‘The Death Poets Society’ by Zahra Zakeri of Queens College, Raymond Birge of Rockefeller University, Richard Lockshin of St. John’s University, Richard Lang of New York University, and Michael Hengartner of Cold Spring Harbor Laboratories. Over the past 29 years, the ICDS has hosted 27 annual international meetings all over the world, as well as satellite meetings in South Africa, Türkiye, Iran, and Brazil to foster collaborations, scientific interchange, and cultural wealth in developing countries. Over the past 30 years, the field of cell death has experienced tremendous ferment and growth, integrating centrally to the fields of developmental biology, immunology, neuroscience, cancer biology, cardiovascular biology, evolutionary biology and others. The biology of cell death has not only witnessed the introduction of new types of cell death (Pyroptosis, Necroptosis, Ferroptosis, Netosis, Panoptosis, Entosis, Autosis, Parthanatos and others) but also the realization of non-cell death related activities of traditional cell death pathways such as Caspases, RIP (Receptor Interacting Protein) kinases, and Gasdermins. ICDS continues to showcase the emerging trends in the fields of cell death and was the primary theme for the 2023 annual meeting.

**Fig. 1:**
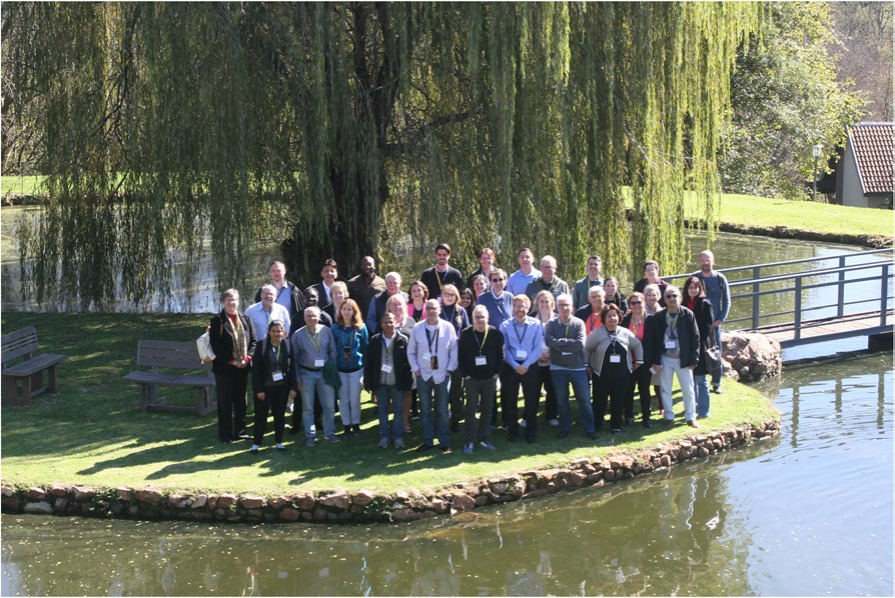
Attending delegates of the 29th ICDS at Glenburn Lodge in South Africa (photograph by Cobus Cronjé)

**Fig. 2:**
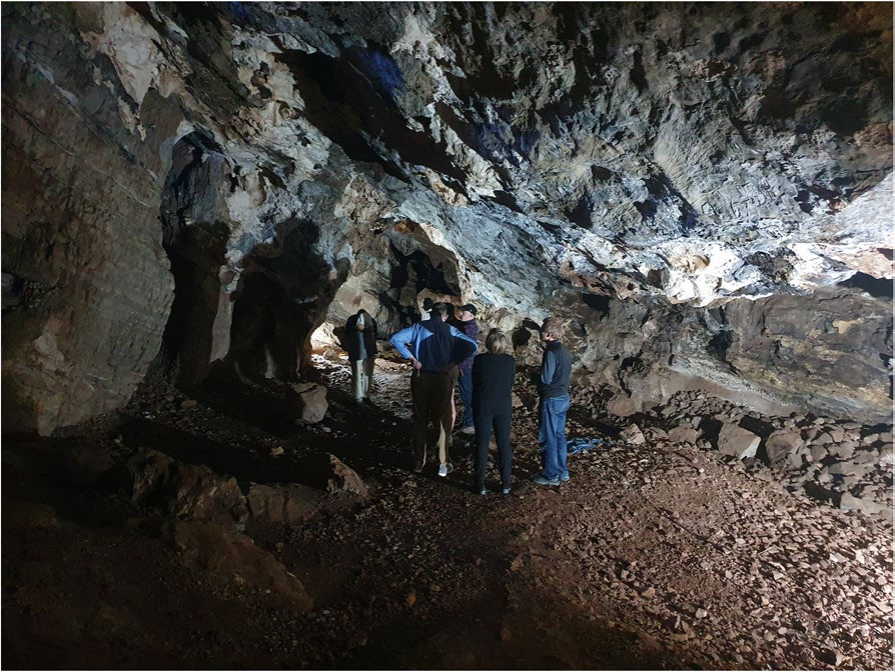
Meeting participants navigated the Sterkfontein limestone caves in Gauteng Province, where some of the earliest human ancestors, hominin fossil remains were discovered (photograph by Cobus Cronjé)

**Fig. 3:**
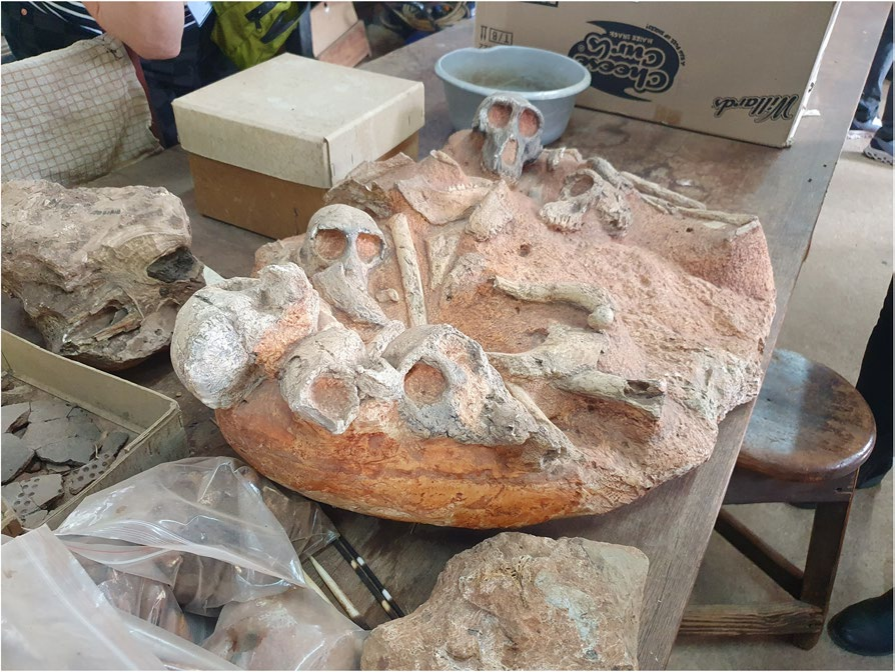
Delegates visiting “The Shed” on site where they could directly interact with technicians who were delicately extracting recently discovered fossils (photograph by Cobus Cronjé)

ICDS’s 29th meeting was held in an exotic location at the Glenburn Lodge in Johannesburg, South Africa under bright African skies (https://guvonhotels.co.za/glenburn-lodge-spa). The meeting had over 25 research talks and numerous posters; and was attended by international delegates representing over 10 different countries (Fig. 1). The meeting benefitted from the support of both Springer Nature (Cell Communication and Signaling) as well as from the University of Witwatersrand, Johannesburg. While the meeting emphasized new trends in Cell Death, the meeting began with a “back to the future” themed onsite tour at the Cradle of Humankind, a historical paleoanthropological site near the meeting site, northwest of Johannesburg in the Gauteng province. Meeting participants navigated limestone caves (Fig. 2), where some of the earliest human ancestors, hominin fossil remains were discovered, and they directly interacted with technicians who were delicately extracting recently discovered fossils (Fig. 3). The Gauteng province caves are some of the oldest paleontologist sites on earth and produced some of the most ancient interments of the hominins.

**Fig. 4:**
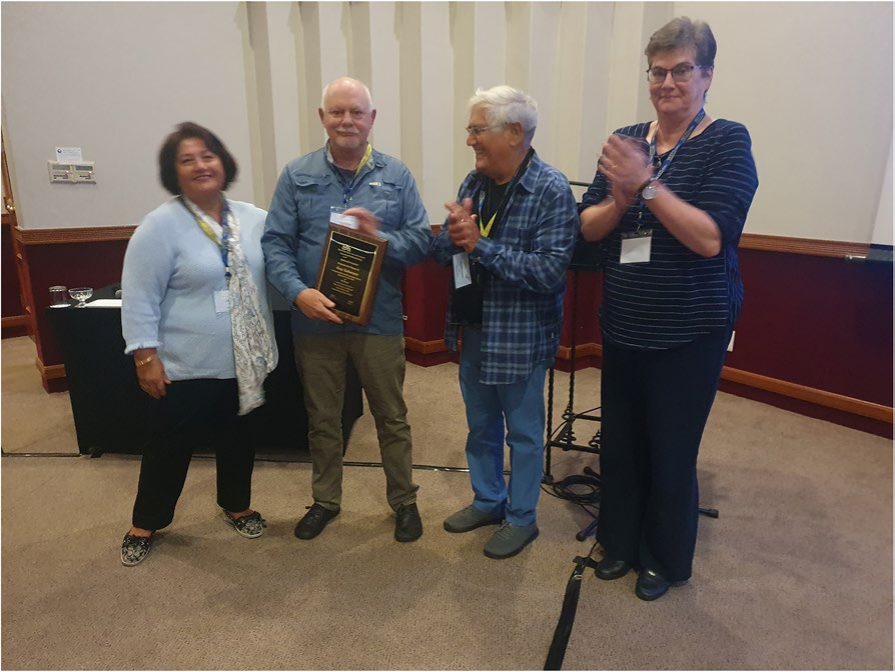
The 2023 ICDS award was presented by Professors Zahra Zakeri (far left), Richard Locksin and Marianne Cronjé (far right) to Professor Guy Salvesen (Burnham Sanford, La Jolla, CA) (photograph by Cobus Cronjé)

The excursion at the cradle of civilization was a fitting start of the scientific sessions and welcoming remarks by Dr. Zahra Zakeri (president of ICDS) and Dr. Marianne Cronjé (co-organizer), and the introduction of the 2023 keynote lecture by Dr. Richard Lockshin. The 2023 ICDS award was presented to Professor Guy Salvesen (Burnham Sanford, La Jolla, CA) (Fig. 4), a biochemist native to South Africa, whose work has greatly contributed to our understanding of caspases and caspase inhibitors (inhibitors of apoptosis or IAPs). Dr. Salvesen discussed the evolution of cell death pathways, particularly those of inflammatory caspases, and likened the downregulation of pathogen-sensing inflammatory caspases to the Red Queen model of evolution, whereby caspases constantly adapt and evolve as their exposure to pathogens as these latter evolve as exemplified by caspase 4; and the Black Queen model of evolution, whereby gene loss is driven by natural selection, for example loss of certain caspase 1 domains in carnivores. Salvesen provided examples where systemic inflammasome downregulation, for example in caspase 5 in some metazoans, can impair host sensing of specific pathogens such that these pathogens can reside undetected in the Carnivora and withstand a cytokine storm. The interesting discussions that ensued addressed the possibility that evolution of new cell death pathways (for example pyroptosis and necroptosis) might emerge from evolutionary adaptations from anti-apoptotic viral and pathogen gene interactions.

Following the award presentations and reception, session 2 (Different models for studying cell death) was chaired by Dr. Marie Hardwick (Johns Hopkins, Baltimore MD) and continued the theme of “back to the future” in cell death research. Dr. Hardwick presented the first talk of the session and emphasized the need for more research on cell death mechanisms in single cell organisms such as bacteria and yeast, where research efforts have historically lagged compared to research on metazoan cell death. Dr. Hardwick acknowledged the existence of Gasdermin-like death in bacteria upon bacteriophage infection, and then discussed a novel vacuolar type of death in heat-shocked and chemically shocked yeast (*Cryptococcus neoformans*). Such vacuolar death is associated with non-mitochondrial permeabilization that may be associated with the release of “Killer cargo” that is reminiscent of, but clearly distinct from, mitochondrial death. The session finished with lectures by Dr. Cornelius Taabazuing (University of Pennsylvania, USA) and Ms. Zaria Malindi (University of Johannesburg, South Africa) who expanded on the theme of inflammatory caspases, with Dr. Taabazuing discussing the specificities of inflammatory caspases (1, 4, and 5 towards IL-1β, IL-18 and Gasdermins) and Ms. Malindi discussing novel antibody drug conjugates that targeted BRAF-driven melanoma by photodynamic therapy and immunogenic cell death.

Session 3 (Different models for studying cell death) was chaired by Professor Sarit Larisch (University of Haifa, Israel), who also presented the first lecture on ARTS (apoptosis-related proteins in the TGF-β signaling pathway) and mitochondrial cell death. Dr. Larisch presented new evidence that ARTS is a master regulator for cell death acting at multiple levels, first by regulating MOMP (mitochondrial outer membrane permeabilization) and the release of SMAC (Second mitochondria-derived activator of caspases) and Cytochrome C from the mitochondria, but also by regulating the degradation of XIAP (X-linked inhibitor of apoptosis protein)and Bcl-2 and by regulating p53. Dr. Larisch emphasized the need for more research in the development of ARTS mimetics, which may act in a manner analogous to that of Venetoclax as a therapeutic. Session 3 proceeded with talks by Dr. Andreas Bergmann (UMASS Medical Center, USA) and Dr. Eli Arama (Weizman Institute of Science, Israel), who discussed the utility of cell death research using *Drosophila melanogaster* as a genetic model. Dr. Bergmann discussed a new pathway for caspase-mediated compensatory proliferation linking DRONC (Drosophila caspase 9) to a novel NADPH oxidase - ROS- TNF - JNK- Fos Jun- pathway. Dr. Bergmann argued that more research should be directed at compensatory proliferation in mammals, particularly related to cancer biology and resistance to drugs. Finally, Dr. Arama finished the session continuing on the theme of non-apoptotic functions of apoptosis in *Drosophila*, emphasizing on both partial organelle destruction, and sperm terminal differentiation, whereby spermatids lose bulk cytosolic content to become mature sperm.

Sessions 4 and 5 (Cell Death and Disease) were co-chaired by Professors Marianne Cronjé (University of the Witwatersrand, South Africa) and Annie Joubert (University of Pretoria, South Africa) and featured themes aimed to target cell death pathways for therapeutic purposes. To begin the session, Dr. Ilia Voskoboinik (Peter MacCallum Cancer Centre, Australia) discussed cytotoxic T cells and Granzyme B killing of target tumor cells and described a mechanism by which externalized phosphatidylserine (PS) on the activated T cells binds perforin and inactivates it, preventing formation of pores, activation of granzyme, and protection against self-killing by the cells’ own perforin. He also emphasized the broader implications for the development of CAR-T’s. Professors Marie Arsenian-Henriksson (Karolinska Institute, Sweden) and Mandeep Kaur (University of the Witwatersrand, South Africa) followed in the session describing vulnerabilities in childhood neuroblastoma by exploiting PRDX6 (Peroxiredoxin 6) and the latter on targeting cholesterol in cancer cells by cyclodextrins, and suppressing epithelial-to-mesenchyme transition (EMT) markers and showing anti-tumor properties (Dr. Kaur). Finally, Dr. Anine Crous (University of Johannesburg, South Africa) discussed methods to improve photodynamic therapies to induce immunogenic cell death in solid tumors including lung, melanoma, and cervical cancer. Day 1 concluded with an interactive poster session while delegates enjoyed sampling local South African wines.

The second day began with two sessions on Cell Death, Disease, and Immunity, co-chaired by Professors Raymond Birge (Rutgers University, USA) and Tugba Bagci-Onder (Koç University, Türkiye) with a series of talks centered on the innate and adaptive immune consequences of dying cells and their clearance by phagocytes. Varsha Gadiyar (Rutgers University, USA) discussed the intrinsic tolerogenic and immune escape consequences of externalized phosphatidylserine on apoptotic and stressed viable cells in the tumor microenvironment, and experimental strategies to target externalized PS and PS scramblases to improve host anti-tumor immunity. Subsequently, Dr. Domagoj Vucic (Genentech, USA) discussed potential therapeutics and the genetics underlying Inflammatory Bowel Disease (IBD), first discussing clinical observations of activation of RIP kinases patients with acute IBD, and that RIP kinase inhibitors can block IBD and TNF-α induced lethal systemic inflammatory response syndrome (SIRS). Dr. Herman Steller (Rockefeller University, USA) expanded the theme on IBD, discussing observations that XIAP deficiency can drive IBD by suppressing inflammasomes, and that XIAP mutations are frequently observed in at-risk individuals for IBD. Dr. Steller also discussed newer data linking ARTS with IBD showing and that small molecule ARTS antagonists (RU2878) can protect from intestinal damage in a chronic colitis DSS (Dextran Sulfate Sodium) model. Next, Dr. Dmitri Krysko (University of Ghent, Belgium) spoke on a potential novel approach for glioma based on immunotherapy and dendritic cells loaded with glioma cells undergoing immunogenic cell death after photodynamic therapy. These strategies using photodynamic therapy efficiently induce immunogenic cell death with some features of ferroptosis. These dying/dead cells release ATP and HMGB1 (high mobility group box 1 protein), which function as adjuvants leading to activation of dendritic cells with subsequent strong anti-tumor T-cell responses. It has been shown that immunogenic cell death and photodynamic therapy based dendritic cell vaccines can be a promising opportunity to efficiently stimulate anti-tumor immunity for enhancing glioma treatment. Finally, Professor Anna Mart Engelbrecht (Stellenbosch University, South Africa) described a compensatory mechanism in the tumor microenvironment whereby chemotherapies such as doxorubicin induce senescent fibroblasts that crosstalk with cancer cells to suppress cell death.

The last two sessions of the meeting, ”Cell Death, Mitochondria, and Cellular Response,” were co-chaired by Dr. Samuel Katz (Yale University, USA) and Dr. Jerry Chipuk (Mount Sinai, USA) and featured a series of talks on the complex roles of mitochondria in cell death, stress, and cancer. Dr. Chipuk started the session discussing BRAF mutations in melanoma and showed that mutant BRAF melanocytes demonstrate a mitochondrial morphology in which the mitochondrial network becomes elongated and expanded, which leads to the activation of the mitochondrial unfolded protein response. At the biochemical level, ATF5 (Activating transcription factor 5) appears to be essential to drive metabolic changes due to proteostasis issues, a response that may be linked to high-risk disease. Dr. Dhyan Chandra (Roswell Park Comprehensive Cancer Centre, USA) continued on the theme of the mitochondrial unfolded protein response in prostate cancer, linking several mitochondrial chaperones and mitochondrial heat shock proteins such as ClpP and HSP60 with malignant features of cancer cells. Both Drs. Chipuk and Chandra echoed a common theme that mitochondrial proteostasis likely underscores a risk for high grade malignant disease.

The theme of mitochondrial cell death and cancer continued in the afternoon session led by a talk by Dr. Shazib Pervaiz (National University of Singapore) who shared the mechanisms of Venetoclax-resistance in Acute Myeloid Leukemia (AML) and other myeloid cancers. Dr. Pervaiz discussed both Bcl-2 and Mcl-1 overexpression as drivers of Venetoclax resistance in AML but also linked drug resistance with redox-mediated phosphorylation of Bcl-2 and Mcl-1, linking mitochondrial ROS (reactive Oxygen Species) with therapeutic responsivity towards Venetoclax. Dr. Marie Veronique Clement (NUS, Singapore) continued the idea of redox regulation and described a pathway by which cells that undergo replicative senescence increase superoxide levels, causing resistance to apoptosis; and she provided a possible explanatio

n for the therapeutic utility for senolytics as novel Bcl-2 inhibitors that target oxidative stress. The session also included an interesting talk by Dr. Samuel Katz (Yale University, USA) describing novel role of BH3 only protein BOK in the regulation of mitochondrial calcium homeostasis and mitochondrial ER contacts, and its role in the type I and III IFN responses to MHV-68 Herpes virus infection. To end the session, Dr. Tugba Bagci-Onder described efforts to develop pathways focused CRISPR screens, called EPIKOL and DDRKOL, to identify novel epigenetic modifiers and DNA damage repair elements which affect chemo- and radio-sensitivity in glioblastoma and induce cell death.

## Concluding remarks

The 29th Annual Scientific Meeting of the ICDS successfully provided cutting edge and exciting dialogue in the now several fields of Cell Death, as well as offered future perspectives in where the fields are going to the next generation. The participants were with the intellectual content of the meeting as well as the warm hospitality and ambience in the conference location of Kromdraai, Mogale City in South Africa. Indeed, many of the delegates went on to post-meeting excursions to the Kruger National Park and other destinations. Excellent science, beautiful locations, warm hospitality and new and old friendships combined to make the 2023 conference a memorable experience. We look forward to the next ICDS meeting in Ghent, Belgium in May 2024 (www.celldeath-apoptosis.org).

